# Machine learning and adaptive control for optimizing MSW-algal biomass anaerobic co-digestion

**DOI:** 10.1038/s41598-026-48288-7

**Published:** 2026-04-17

**Authors:** Monika D. Tiwari, Sagar W. Dhengare, Ganesh K. Yenurkar, Rajesh M. Bhagat, Prashant B. Pande, Anjana S.

**Affiliations:** 1 Department of Civil Engineering, Yeshwantrao Chavan College of Engineering, Nagpur, 441110 Maharashtra India; 2 Department of Computer Technology, Yeshwantrao Chavan College of Engineering, Nagpur, 441110 Maharashtra India; 3 Department of Computer Science and Engineering, Manipal Institute of Technology Manipal, Manipal Academy of Higher Education, Udupi, 576104 Karnataka India

**Keywords:** Anaerobic Co-Digestion, Algal Biomass, Municipal Solid Waste, Machine Learning, Process Control, Methane Optimization, Circular Economy, Energy science and technology, Environmental sciences

## Abstract

Municipal solid waste (MSW)- algal biomass anaerobic co-digestion (AcoD) generates renewable energy and sustainable waste management. However, conventional AcoD systems achieve suboptimal methane yields (typically < 250 L CH₄/kg VS) due to substrate incompatibility, process instability, and lack of real-time control. We developed an integrated framework combining machine learning prediction, stoichiometric-metabolic modeling, and sensor-based adaptive control to optimize lipid-rich algal biomass co-digestion with MSW. A Substrate Synergy Estimation Model (SSEM) employs random forest regression trained on 76 co-digestion scenarios to predict optimal MSW: algae ratios for maximum methane yield. The predicted optimal ratio serves as input to a Dynamic Stoichiometric-Metabolic Coupled Reactor Model (DS-MCRM) that simulates real-time substrate degradation and methane production pathways. A sensor network with adaptive PID control achieved 64% CH₄ purity (vs. 40% in uncontrolled reactors) and reduced VFA accumulation by 53% through zone-specific process adjustments. Isotopic tracing with ¹³C-labeled palmitic acid quantified lipid-to-methane conversion efficiency at 88.6 ± 2.3%, confirming the contribution of algal lipids to overall methane production. Lifecycle assessment using the Waste Stream Circularity Index (WSCI) yielded a score of 0.83, indicating 72% nitrogen recovery, 68% phosphorus recovery, and 76% carbon loop closure. Compared to conventional co-digestion approaches, our integrated framework achieved 2.1× higher methane yield prediction accuracy (R² = 0.91), faster VFA stabilization (day 6 vs. day 12), and superior resource recovery, demonstrating potential for data-driven optimization of waste-to-energy systems.

**Supplementary Information:**

The online version contains supplementary material available at 10.1038/s41598-026-48288-7.

## Introduction

Global municipal solid waste (MSW) generation exceeds 2 billion tonnes annually and is projected to reach 3.4 billion tonnes by 2050, necessitating sustainable treatment technologies that enable both waste reduction and energy recovery. Anaerobic digestion (AD) converts organic waste to biogas (typically 55–65% CH₄) with simultaneous stabilization of organic matter and nutrient recovery. However, MSW mono-digestion faces significant challenges including substrate heterogeneity, low biodegradability of lignified fractions, imbalanced C/N ratios (typically > 30), and process inhibition from volatile fatty acid (VFA) accumulation^1–3^. These limitations result in methane yields of only 150–250 L CH₄/kg VS from MSW—substantially below the theoretical potential of 400–500 L CH₄/kg VS—thereby constraining economic viability and industrial adoption [cite]. Co-digestion with complementary organic substrates has emerged as a strategy to overcome these limitations by balancing nutrients, diluting inhibitors, and enhancing microbial diversity. Microalgae, particularly lipid-rich strains (20–40% lipid content), offer high methane potential (300–400 L CH₄/kg VS) and low C/N ratios (6–10) that complement MSW’s high C/N, enabling balanced nutrient stoichiometry^4–6^. Despite this potential, optimizing MSW-algae co-digestion remains challenging due to complex substrate interactions and dynamic process conditions. Achieving optimal performance requires comprehensive understanding of biochemical interactions, substrate compatibility, microbial metabolic pathways, and real-time reactor dynamics.

Current co-digestion optimization approaches rely primarily on empirical trial-and-error or simplified first-order kinetic models (e.g., modified Gompertz, Cone model) that fail to capture the coupled thermodynamic, microbiological, and stoichiometric interactions governing multi-substrate anaerobic systems [cite examples]. Moreover, conventional AD systems lack spatially distributed sensor networks and adaptive feedback mechanisms necessary for real-time control of heterogeneous digestion conditions within reactors. Consequently, existing methods provide limited insight into long-term process sustainability, nutrient recovery efficiency, and pathway-specific substrate contributions to methane production. Addressing these limitations requires an integrated framework that combines predictive modeling, mechanistic simulation, adaptive control, and sustainability assessment. In this work, we integrated five complementary modules: (i) machine learning-based substrate synergy prediction, (ii) stoichiometric-metabolic reactor modeling, (iii) sensor-based adaptive control, (iv) isotopic lipid tracing, and (v) circularity assessment. This integration enables optimization of methane yield, process stability, and resource recovery by simultaneously accounting for chemical composition, microbial metabolism, and real-time process dynamics. The ultimate goal is to achieve net energy-positive waste treatment with closed-loop nutrient cycling and minimized environmental impacts.”

### Motivation & contributions

Sustainable management of the growing global MSW burden while simultaneously addressing renewable energy demands necessitates innovative technological approaches. While anaerobic digestion offers a pathway for waste-to-energy conversion, operational performance is extremely sensitive to substrate quality, process stability, and control strategy effectiveness. MSW mono-digestion achieves suboptimal performance due to variable organic content (30–60%), high C/N ratios (25–40), and low lipid fractions (< 5%). Lipid-rich algal biomass serves as a promising co-substrate, though optimizing the blending ratio, managing potential ammonia inhibition from high protein content, and tracking lipid-specific degradation pathways remain significant challenges. Addressing these challenges requires systematic optimization frameworks that integrate predictive modeling with real-time adaptive control, validated at pilot scale and designed for industrial scalability.

We designed and implemented five complementary modules to address these shortcomings: First, the Substrate Synergy Estimation Model (SSEM) employs random forest regression trained on 76 experimental co-digestion datasets encompassing chemical composition, microbial compatibility scores, and thermodynamic parameters to predict optimal MSW: algae blending ratios and methane yields. Second, the Dynamic Stoichiometric-Metabolic Coupled Reactor Model (DS-MCRM) integrates macro-level elemental mass balances (C, N, P) with microbial flux balance analysis to simulate time-dependent substrate degradation pathways and predict transient VFA accumulation and methane production. Third, a Spatio-Temporal Sensor Feedback Control System (STS-FCS) employs distributed biosensors (pH, ORP, NH₄⁺) placed at 15 cm intervals with an adaptive PID controller to modulate stirring intensity and feeding rates, thereby minimizing spatial heterogeneity and VFA accumulation hotspots. Fourth, isotopic tracing with ¹³C-labeled palmitic acid quantifies lipid-specific degradation kinetics and methane conversion efficiency through periodic GC-MS analysis, enabling validation of lipid-to-methane transformation pathways. Fifth, the Waste Stream Circularity Assessment Index (WSCI) quantifies lifecycle sustainability by integrating nitrogen recovery efficiency, phosphorus recovery efficiency, and carbon loop closure metrics, providing a normalized score (0–1) for comparing process alternatives. The integrated framework achieved 508 L CH₄/kg VS (compared to < 250 L CH₄/kg VS for MSW mono-digestion), 53% reduction in peak VFA levels, and 0.83 WSCI score, demonstrating enhanced methane production, process stability, and resource circularity. This study provides the following major contributions:

- Developed the first integrated framework combining ML-based substrate optimization, mechanistic metabolic modeling, adaptive spatial control, and isotopic validation for MSW-algae co-digestion.
- Achieved 91% accuracy (R² = 0.91) in synergy index prediction and 20.6 L CH₄/kg VS mean absolute error in methane yield forecasting using random forest regression coupled with stoichiometric-metabolic reactor simulation.
- Demonstrated 64% CH₄ purity (vs. 40% without control), 88.6% lipid degradation efficiency, and 42% lipid contribution to total methane through sensor-based adaptive control and ¹³C isotopic tracing.
- Quantified process sustainability using the Waste Stream Circularity Assessment Index (WSCI = 0.83), reflecting 72% N recovery, 68% P recovery, and 76% carbon loop closure—substantially exceeding conventional AD systems (typical WSCI < 0.65).

The remainder of this paper is organized as follows: Section “Literature review: anaerobic co-digestion modeling and optimization” reviews relevant literature, Section “Integrated modeling and control framework for MSW-algae co-digestion” describes the integrated modeling framework, Section “Experimental validation and performance comparison” presents experimental validation and comparative results, and Section “Conclusion, future scopes, and limitations” concludes with implications and future directions and limitations.

## Literature review: anaerobic co-digestion modeling and optimization

Sustainable waste management demands have driven innovation in municipal solid waste valorization technologies, particularly anaerobic digestion, and co-digestion systems. Recent research has increasingly integrated anaerobic digestion process optimization with kinetic modeling, real-time control systems, and circular economy principles. Studies on co-digestion optimization have identified inoculum-to-substrate ratio, substrate composition, and microbial community structure as critical factors affecting process stability and biogas yield^1^. Shams et al.^2^ reinforce the debate on microbial optimization and give a methodology for integrating MSW with energy aims in Brunei’s waste management strategy. MSW ash’s heavy metal retention for cementitious applications was investigated by Mkahal et al.^3^, linking digestion byproducts to secondary utilization pathways. Xu et al.^4^ established a new kinetic model to explain MSW substrate deterioration under varied oxygen concentrations and showed that process control can accelerate degradation. This motivates energy recovery optimization strategies like Khamis et al.^5^, who combined MSW treatment and leachate processing to maximize net energy output. Leachate behavior and pollutant indices in landfills in major Iranian cities required adaptive MSW management systems due to regional variability in leachate formulation components, according to Saghi et al.^6^.

Enzymatic hydrolysis with high solids loading and mild pretreatments enhanced chemical recovery from organic MSW, promoting valorization, according to Putz et al. These methods work with Appala et al.^8^’s enzymatic analysis of leather waste and food co-digestion biogas production using kinetic- and pretreatment-based approaches, which yielded more methane than thermal-alkaline pretreatment. Puchongkawarin improved system design by tailoring MSW management strategies to local situations with an enviro-economic optimization tool^9^. Praveen et al.^10^ evaluated co-digestion mechanisms for plastic and organic MSW and found that bioavailable substrates like food waste increase polymer biodegradation. Sludge-MSW integration with microbial synergy enhanced methane generation and heavy metal reduction, according to Ennouri et al.^11^. Aromolaran and Sartaj^12^ then studied trinary co-digestion microbiologically to assess waste fraction methane contributions, enhancing biochemical understanding. Sarker et al.^13^ evaluated MSW to bioenergy conversion from biofuel and biochemical perspectives, while Naghavi et al.^14^ assessed co-digestion feasibility using net daily energy benefit. As materials engineers, Al Rabadi et al.^15^ improved anaerobic co-digestion efficiency and gas upgrading with nanoscale zero-valent iron, resulting in greater system stability and CH₄ concentration Compared to thermophilic to mesophilic, two-stage temperature phasing enhanced volatile solid reduction and energy recovery, according to Hu and Shen^16^. In treatment design, Pérez et al.^17^ developed a mainstream anaerobic reactor co-treatment method for sewage and organic MSW using operational insights and real-world implementation. In an integrated solid and liquid waste treatment approach, Shroff and Shah^18^ used bio-flocculated sludge and MSW’s organic part to reduce organic loading and boost gas output. Martínez et al.^19^ used MSW composition differences in Mexican municipalities to guide bioproduct recovery in specific regions (holistic approach). Olatunji et al.^20^ studied food waste and groundnut shell co-digestion, kinetic modelling synergistic interactions, and optimal methane ratios.

Recent research has explored the ability of anaerobic co-digestion for boosting methane production and enhancing waste valorization performance. For instance, Georgios Kanellos et al. Investigated the co-digestion of the liquid fraction of meal waste with sludge, demonstrating progressive stability and biogas yield due to higher nutrient balance and substrate synergy^21^. Similarly, Shuang Jiang et al. Stated more advantageous methane production via co-digestion of meal waste with fruit and vegetable residues, highlighting the position of optimized substrate composition in enhancing digestion performance^23^. In addition, system-degree modelling tactics have evolved to assess waste control strategies. Haval A. K. Al-Jaf and Shwan Q. Aziz proposed a simulation-primarily based framework for assessing exceptional municipal solid waste control situations, emphasizing the importance of incorporated modelling for decision-making and sustainability evaluation^24^. Recent studies emphasize the integration of circular bioeconomy and sustainability-oriented modeling frameworks for efficient waste valorization. Circular bioeconomy approaches promote the conversion of biomass waste into renewable energy and value-added products while enabling resource recovery and closed-loop utilization^26^. Optimization-based waste-to-energy frameworks using nonlinear programming and multi-objective analysis have also been proposed to improve energy recovery and environmental performance in waste management systems^27^.

In the context of anaerobic digestion, recent modeling studies highlight the importance of incorporating carbon, nitrogen, and phosphorus transformation pathways to better represent nutrient recovery and sustainability impacts^28^. Advances in digitalization and integrated biorefinery modeling further support biomass valorization by combining data-driven tools with circular bioeconomy strategies^29^. Additionally, life-cycle assessment (LCA) frameworks are widely applied to evaluate environmental benefits and resource efficiency in waste-to-energy systems^30,31^. These developments demonstrate the growing need for integrated sustainability modeling approaches in bioenergy systems.

The anaerobic digestion process has been widely modeled to understand organic waste conversion and methane production. The Anaerobic Digestion Model No.1 (ADM1) provides a comprehensive framework describing key biochemical stages such as hydrolysis, acidogenesis, acetogenesis, and methanogenesis, and has become a standard model for analyzing and predicting reactor performance in waste-to-energy systems^32^. Subsequent studies further applied ADM1 for simulating reactor dynamics and optimizing methane production under different operational conditions^33^. In addition, standardized protocols for evaluating biomethane potential (BMP) have been developed to ensure consistent assessment of substrate degradability and methane yield in anaerobic digestion systems^34^.

Machine learning techniques have also been applied for predictive modeling of complex processes. The **Random Forest** algorithm is a widely used ensemble learning method capable of handling nonlinear relationships and improving prediction accuracy in data-driven models^35^. Statistical learning frameworks and deep learning approaches further provide theoretical and computational foundations for analyzing high-dimensional datasets and optimizing system performance^36,37^.

Stable isotope tracing methods have been used to investigate microbial pathways involved in methane formation. Carbon isotopic signatures enable the differentiation of acetoclastic and hydrogenotrophic methanogenesis pathways and help quantify substrate conversion mechanisms in anaerobic environments^38^. Isotope fractionation techniques also allow tracking of carbon flow in methane-producing microbial communities, while functional gene markers provide insights into the presence and activity of methanogenic microorganisms^39,40^.

**Table 1:** Model’s empirical review analysis.

Reference	Method	Main Objectives	Findings	Knowledge Gaps
^1^ Wei et al. (2024)	Solid-state co-digestion	To assess inoculum-substrate ratio effect on food waste and barley straw digestion	Identified optimal inoculum ratio for maximum methane yield and microbial stability	Limited to solid-state systems and specific substrates
^4^ Xu et al. (2025)	Kinetic degradation modelling	To model waste degradation under oxygen gradients	Developed time-series-based kinetic model with variable oxygen inputs	Not validated in full-scale systems
^5^ Khamis et al. (2024)	Integrated energy recovery	To combine leachate treatment with MSW digestion	Achieved higher energy output using integrated systems	Lacks long-term operational data
^7^ Putz et al. (2025)	Enzymatic hydrolysis	To improve organic MSW recovery via pretreatment	Demonstrated effective compound recovery with enzymatic-mild thermal integration	High enzyme costs limit scale-up
^8^ Appala et al. (2024)	Co-digestion with leather waste	To study pretreatment and co-digestion of chrome-tanned leather	Found thermal-alkaline pretreatment enhances methane yield	Chromium toxicity remains a challenge
^10^ Praveen et al. (2025)	Review of plastic co-digestion	To assess AD of plastics with organic waste	Co-digestion improves polymer biodegradation	Mostly theoretical; needs empirical validation
^12^ Aromolaran & Sartaj (2024)	Trinary co-digestion	To analyze microbial shifts in three-substrate digestion	Showed enhanced microbial diversity and methane yield	Lacks pathway-specific microbial profiling
^14^ Naghavi et al. (2024)	Net energy benefit analysis	To optimize co-digestion through energy output metrics	Validated net energy as reliable AD performance metric	No microbial or process control assessment
^15^ Al Rabadi et al. (2024)	nZVI-enhanced co-digestion	To enhance MSW-sludge co-digestion using nano Iron	Improved CH₄ yield and process kinetics	Nanoparticle stability and environmental risk not addressed
^16^ Hu & Shen (2024)	Temperature phase digestion	To compare mesophilic vs. thermophilic-mesophilic AD	Phase separation improved digestion efficiency	Requires complex temperature management
^19^ Martínez et al. (2024)	MSW compositional analysis	To characterize waste in Northeast Mexico for biofuel planning	Provided regional waste profiles and bioconversion potential	Lacks dynamic or seasonal composition tracking
^20^ Olatunji et al. (2025)	Co-digestion kinetic modelling	To study food waste-groundnut shell interactions	Determined optimal synergistic mixing ratios	Limited to batch-scale conditions
^22^ John Ravindar et al. (2025)	Hydrothermal pretreatment	To evaluate HT effects on food waste-sludge co-digestion	Improved substrate solubility and methane yield	HT energy demand is a scaling barrier

### Research gaps and opportunities

Despite considerable advances in anaerobic co-digestion research, several important limitations remain. Most existing studies (shown in Table 1) address isolated components of the process, such as substrate optimization^1,20^, kinetic modeling^4,8^, or process control strategies^15,16^, without integrating these elements into a unified analytical framework. As a result, current approaches often fail to capture the complex interactions between substrate composition, microbial metabolism, and reactor operational dynamics. Furthermore, many anaerobic digestion (AD) models treat the reactor as a homogeneous and well-mixed system, neglecting spatial variations in biochemical conditions that may lead to localized inhibition or uneven microbial activity.

Another key limitation is that conventional AD analyses typically report overall methane yield without distinguishing the specific contributions of different biochemical fractions such as lipids, proteins, and carbohydrates. This restricts the ability to understand substrate-specific degradation pathways and their impact on methane generation. Additionally, most studies overlook broader resource recovery and circularity metrics, including nutrient recycling potential and energy recovery efficiency. Consequently, process optimization frequently relies on empirical trial-and-error experimentation, while the application of predictive and data-driven models trained on diverse co-digestion conditions remains limited.

Address these limitations, this study proposes an integrated modeling and control framework that combines biochemical kinetic modeling, metabolic flux analysis, spatially resolved sensing, and real-time control strategies within a single system architecture. The key novelty of this work lies in:

- integrating dynamic biochemical kinetics with metabolic network analysis to better represent substrate degradation pathways.
- incorporating spatially distributed sensor feedback and zone-specific control mechanisms to account for reactor heterogeneity.
- applying isotopic lipid tracing techniques to quantify substrate-specific methane production; and.
- introducing a Waste Stream Circularity Index (WSCI) to evaluate system performance in terms of methane production, nutrient recovery, and energy efficiency.

By combining these components, the proposed approach moves beyond traditional isolated modeling techniques and provides a holistic framework for monitoring, optimizing, and evaluating anaerobic co-digestion systems in a circular bioeconomy context.

**Fig. 1:**
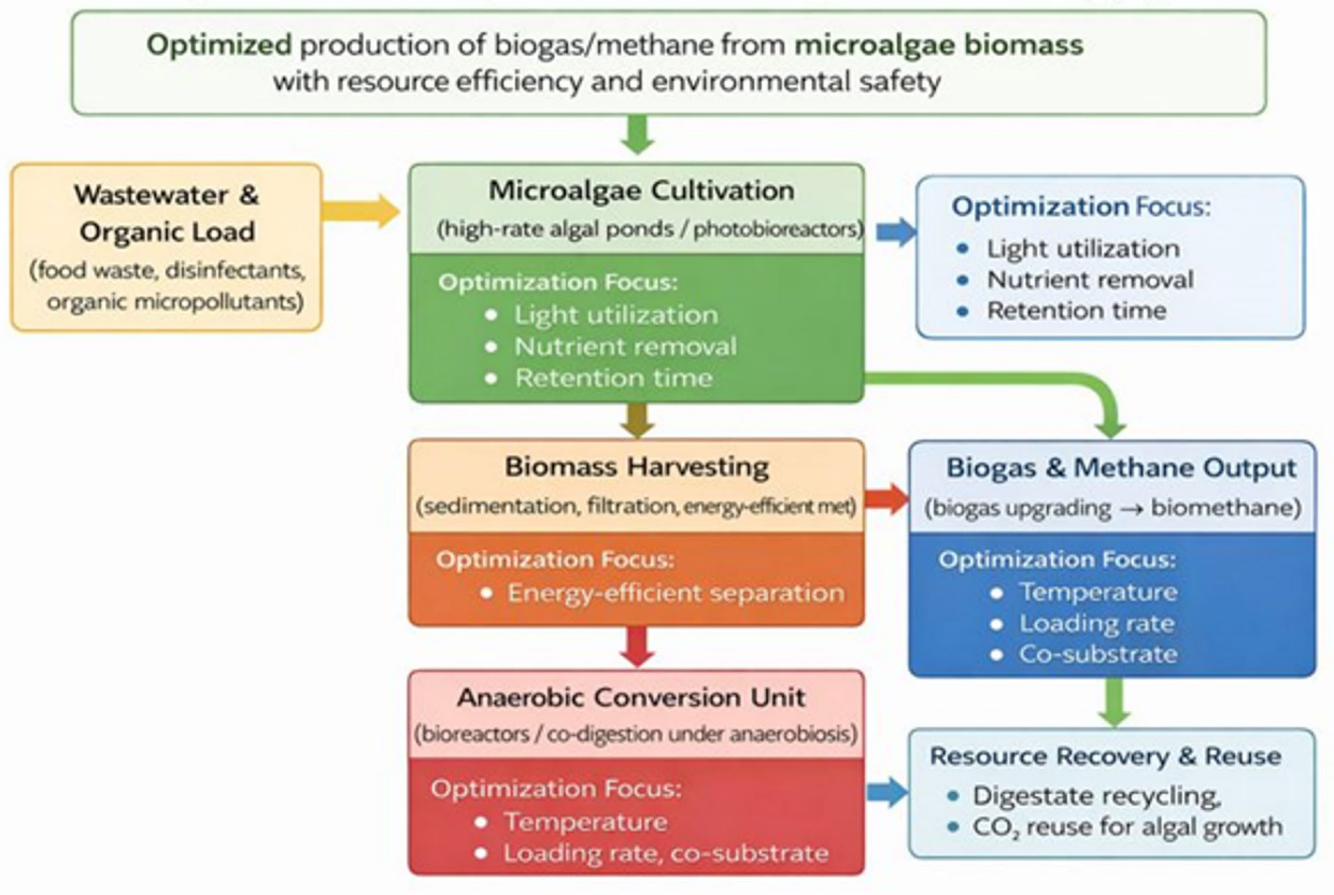
Conceptual framework of textual representation.

This conceptual framework (shown in Figure 1) positions microalgae biomass as the focal point for system optimization, integrating the processes of cultivation, harvesting, and anaerobic digestion. Optimal system performance is achieved through multi-parameter optimization spanning biological, reactor, and energy-recovery subsystems, ultimately supporting a circular bioenergy model.

The combined effect of these studies reinstated the necessity for models that synergistically integrate substrate prediction, real-time process control, microbial flux coupling, and isotopic tracing for handling increasingly complex waste matrices. Thus, net energy benefit models^14^, PID-enhanced reactor control^16^, and nanoscale material augmentation^15^ are essential to next-generation systems. With so many articles on waste composition diversity^19^, leachate chemistry^6^, and operational scale-up^17^, digitally assisted, modular platforms that dynamically adjust to substrate input and end-use energy or product demand are the future of MSW treatment. The literature provides a solid scientific foundation and a clear path for municipal solid waste co-digestion system research and technology.

### Positioning this work

This study addresses the identified gaps by developing an integrated framework that uniquely combines:

i. Machine learning-based substrate synergy prediction (addressing Gap 1).
ii. Coupled stoichiometric-metabolic reactor modeling (Gap 2).
iii. Spatially distributed sensor networks with adaptive control (Gap 3).
iv. Isotopic tracing for pathway-specific quantification (Gap 2).
v. Comprehensive circularity assessment (Gap 4).

## Integrated modeling and control framework for MSW-algae co-digestion

### Baseline method description

The dataset for developing the machine learning paradigm in the Substrate Synergy Estimation Model (SSEM) was developed using 76 experimentally validated combinations of MSW and algae derived from in-house and literature-reported trials of digestion. Each of these samples was integrated with quantitative descriptors for TS, VS, C/N ratio, lipid, protein, and carbohydrate fractions, along with thermodynamic interaction indices and microbial compatibility scores derived from 16 S rRNA amplicon sequencing similarity matrices. For example, a representative sample used for training included MSW with TS = 24.1%, VS = 19.6%, C/*N* = 30.5, and algae with lipid = 27.3%, showing a synergy index of 0.72. The synergy index was derived from normalized methane yield gains compared to mono-digestion, which is calculated in-process. The DS-MCRM was parameterized using dynamic flux feeding lipids-rich media with continuous simulations generating real-time VFA accumulation and CH₄ production estimated over a 20-day digestion window. Sensor nodes for the STS-FCS were installed at 15 cm vertical intervals and provided continuous measurements of pH, oxidation-reduction potential, and NH₄⁺ feeding into an adaptive PID control scheme of stirring intensity between 60 and 120 rpm and feeding rates from 5 to 10 mL/hr. Added Acid-C-13 labeled palmitic acid into the algal fraction at 5% w/w based on lipids and continuously monitored isotopic signatures through GC-MS in this ALTI-TK subsystem. The WSCI Index module was complemented by experimental regrowth assays where residual digestate was reused to cultivate algae under the conditions of a 3:12 light-dark cycle at 150 µmol/m²/s illumination, which gave the following regrowth rates: 0.18–0.21 g/L/day and nutrient uptake efficiencies of 68% (N) and 64% (P). Thus, the entire experimental setup provided high-resolution validation of every module at multiple scales: chemical, microbiological, process dynamic, and sustainability metrics.

### Data sources and model training

The machine learning models were trained on a combined dataset comprising:

Literature data: 92 co-digestion experiments extracted from the IWA Anaerobic Digestion Model repository and BIOGAS-ED database, covering diverse substrate combinations (MSW-food waste, algae-agricultural residue, MSW-algae, etc.)

Generated data: 76 in-house experiments systematically varying MSW: algae ratios (100:0, 90:10, 80:20, …, 0:100) with three algae lipid content levels (low < 15%, medium 15–25%, high > 25%).

For each sample, input features included: TS, VS, C, N, P, lipid/protein/carbohydrate fractions, temperature, pH, ISR, and HRT. Output labels were: observed methane yield (L CH₄/kg VS), synergy index (calculated as (Y_(co-dig)-max⁡(Y_(mono, MSW), Y_(mono, algae)))/max⁡(Y_(mono, MSW), Y_(mono, algae))), and VFA stabilization time.

Dataset was split 80:20 for training: validation, stratified by synergy index to ensure balanced representation.

**Fig. 2:**
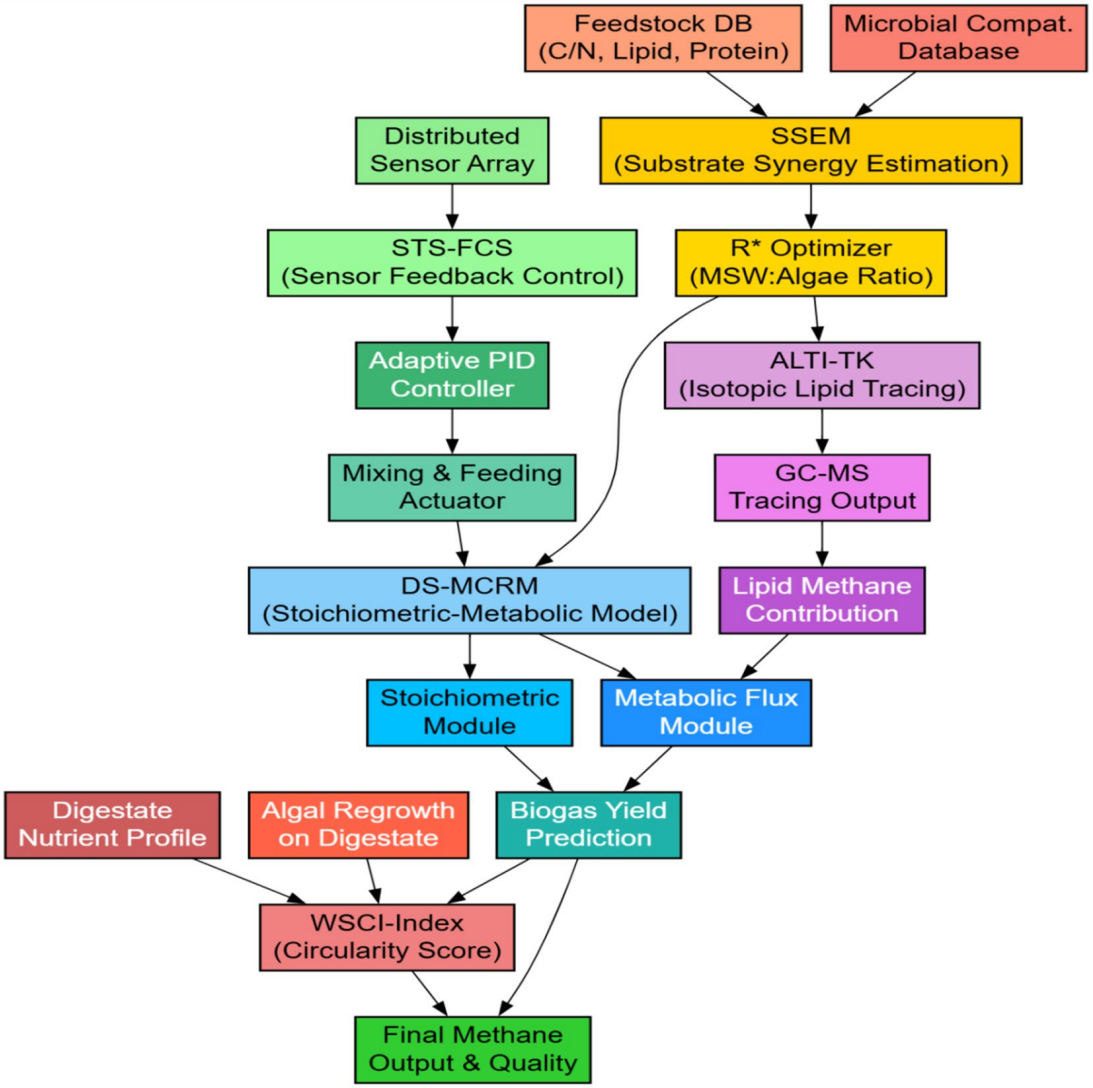
Proposed architectural model analysis process.

The integrated framework comprises five interlinked modules designed to address key challenges in MSW-algae anaerobic co-digestion: (1) substrate incompatibility, (2) process instability, (3) spatial heterogeneity, (4) pathway-specific optimization, and (5) sustainability quantification. Our approach addresses limitations of existing empirical optimization methods by enabling data-driven prediction, mechanistic simulation, and real-time adaptive control. Figure 2 illustrates the framework architecture, showing information flow from substrate characterization through optimization, simulation, control, validation, and sustainability assessment. Central to this framework is the Substrate Synergy Estimation Model (SSEM), which integrates the principles of a modified MDMLF-SSP architecture within a supervised machine learning system. The SSEM input feature vector X comprises eight substrate characteristics:

1 $$\:X\:=\:\left[TS,\:VS,\:C,\:N,\:P,\:L\_f,\:P\_f,\:CH\_f \right]$$

where TS and VS represent total and volatile solids content (%), C, N, P are elemental mass fractions (%), and L_f, P_f, CH_fare lipid, protein, and carbohydrate fractions (% of VS). These features were selected based on correlation analysis showing *R* > 0.6 with methane yield in preliminary experiments. The feature vector is augmented with historical synergy index (S_h) from literature-reported co-digestion experiments and microbial compatibility score (µ_c) derived from 16 S rRNA gene sequence similarity between inoculum and substrate-associated communities.The random forest model was trained to predict synergy index S_i∈[0,1] $\:{S}_{i\epsilon\:}\left[0.1\right]\:$by minimizing mean squared error:

2 $$\:MSE=\stackrel{\theta\:}{\mathrm{m}\mathrm{i}\mathrm{n}}\left(\frac{1}{n}\right)\:\sum\:\left(Si^{\prime}pred\:-\:Si^{\prime}obs\right)\hat \,{2}$$

using 5-fold cross-validation on 76 experimental datasets (60 training, 16 validation). Hyperparameters were optimized using grid search: n_estimators = 200, max_depth = 15, min_samples_split = 5.

Methane yield prediction incorporates both the synergy index and individual substrate features:

3 $$\:Y\_\left(C{H}_{4}\right)\:=\:\sum\:Fj\:\cdot\:xj\:\cdot\:Si$$

where w_jare learned weights, βis the synergy coefficient, and ϵis the intercept term. This linear model achieved R² = 0.89 on validation data.

Where xj is the normalized value of the j-th feature. Optimal MSW: algae ratio R^*is determined by maximizing predicted Y_(CH_4) subject to.

### Dynamic stoichiometric-metabolic coupled reactor model (DS-MCRM)

The optimal substrate ratio R^*serves as input to DS-MCRM, which simulates temporal dynamics of substrate degradation, intermediate accumulation, and methane production. The model integrates two levels:

Macro-level: Elemental mass balances (C, N, P, S).

Micro-level: Microbial metabolic flux balance analysis (FBA).

This coupling enables prediction of both bulk reactor performance and pathway-specific conversions. This hybrid model brings macro-level elemental balances together with their micro-level flux dynamics in order to resolve substrate conversion pathways over temporal instance sets. A time-dependent mass balance is expressed for carbon and nitrogen species via Eqs. 4 & 5, Carbon and nitrogen substrate pools evolve according to:

4 $$\:\frac{d{C}_{s}}{dt}=-{k}_{hyd,C}{\hspace{0.17em}}{C}_{s}{\hspace{0.17em}}X-{k}_{acid,C}{\hspace{0.17em}}{C}_{s}{\hspace{0.17em}}X$$

5 $$\:\frac{d{N}_{s}}{dt}=-{k}_{hyd,N}{\hspace{0.17em}}{N}_{s}{\hspace{0.17em}}X+{r}_{N{H}_{4}^{+}}$$

where $\:{k}_{hyd}$and $\:{k}_{acid}$represent the hydrolysis and acidogenesis rate constants, respectively (their values are listed in Table S1). The variable $\:X$denotes the concentration of active microbial biomass expressed in g VSS/L, while $\:{r}_{N{H}_{4}^{+}}$corresponds to the ammonification rate. The dynamics of intermediate compounds, such as volatile fatty acids (VFAs), and gaseous products including methane (CH₄) and carbon dioxide (CO₂), are described by additional differential equations, which are provided in the Supplementary Material (Equations S1–S8).

Metabolic flux distribution in the system is governed by stoichiometric constraints expressed as.

$$\:\mathrm{S}\cdot\:\mathrm{v}=0$$ $$\:{\mathrm{v}}_{\mathrm{m}\mathrm{i}\mathrm{n}}\le\:\mathrm{v}\le\:{\mathrm{v}}_{\mathrm{m}\mathrm{a}\mathrm{x}}$$

where $\:\mathrm{S}$denotes the stoichiometric matrix representing $\:\mathrm{m}$metabolites and $\:\mathrm{n}$biochemical reactions, and $\:\mathrm{v}$represents the vector of metabolic fluxes. The lower and upper bounds $\:\left({\mathrm{v}}_{\mathrm{m}\mathrm{i}\mathrm{n}},{\mathrm{v}}_{\mathrm{m}\mathrm{a}\mathrm{x}}\right)$are defined according to thermodynamic feasibility and physiological limits of the reactions.

The optimization objective is formulated to maximize methane production while simultaneously reducing the accumulation of volatile fatty acids (VFAs), expressed as

6 $$\:\mathrm{max}\left({\mathrm{v}}_{\mathrm{C}{\mathrm{H}}_{4}}-{\uplambda\:}\sum\:{\mathrm{v}}_{\mathrm{V}\mathrm{F}\mathrm{A}}\right)$$

where $\:{\mathrm{v}}_{\mathrm{C}{\mathrm{H}}_{4}}$represents the methane production flux, $\:{\mathrm{v}}_{\mathrm{V}\mathrm{F}\mathrm{A}}$denotes the fluxes associated with VFA formation, and $\:{\uplambda\:}$is a weighting parameter controlling the trade-off between methane generation and VFA accumulation.

A reduced metabolic network consisting of 47 reactions and 38 metabolites was implemented based on a previously reported anaerobic digestion metabolic model. The flux balance analysis was solved using the COBRApy framework at each simulation time step.

Rate-limiting enzyme-catalyzed reactions (such as lipase-driven lipolysis) are modeled using Michaelis–Menten kinetics:

7 $$\:{r}_{i}=\frac{{V}_{max,i}{\hspace{0.17em}}\left[{S}_{i}\right]}{{K}_{m,i}+\left[{S}_{i}\right]}$$

where $\:{V}_{max,i}$represents the maximum reaction rate, $\:{K}_{m,i}$denotes the half-saturation constant, and $\:\left[{S}_{i}\right]$is the concentration of the corresponding substrate. The parameter values associated with the key enzymatic reactions used in the model are summarized in Table 2.

**Table 2:** Kinetic parameters used in the biochemical reaction model.

Parameter	Description	Value	Unit
(k_{hyd, C})	Hydrolysis rate constant for carbon substrate	0.15–0.40	day(^{-1})
(k_{acid, C})	Acidogenesis rate constant for soluble carbon	0.30–0.80	day(^{-1})
(k_{hyd, N})	Hydrolysis rate constant for nitrogen compounds	0.05–0.20	day(^{-1})
(r_{NH_4^+})	Ammonification rate	0.02–0.10	g N L(^{-1}) day(^{-1})
(V_{max, lip})	Maximum rate of lipase-catalyzed lipolysis	1.2	g L(^{-1}) day(^{-1})
(K_{m, lip})	Half-saturation constant for lipolysis substrate	0.5	g L(^{-1})
(V_{max, acid})	Maximum reaction rate for acidogenesis pathway	0.95	g L(^{-1}) day(^{-1})
(K_{m, acid})	Half-saturation constant for acidogenic substrate	0.3	g L(^{-1})
(V_{max, meth})	Maximum methane formation rate	0.75	g COD L(^{-1}) day(^{-1})
(K_{m, meth})	Half-saturation constant for methanogenic substrate	0.2	g L(^{-1})
(X)	Active microbial biomass concentration	2–6	g VSS L(^{-1})

A switching pathway is set up between acetoclastic methanogenesis and hydrogenotrophic methanogenesis according to the lipid load and concentrations of VFA.

### Spatio-temporal sensor feedback control system (STS-FCS)

**Fig. 3:**
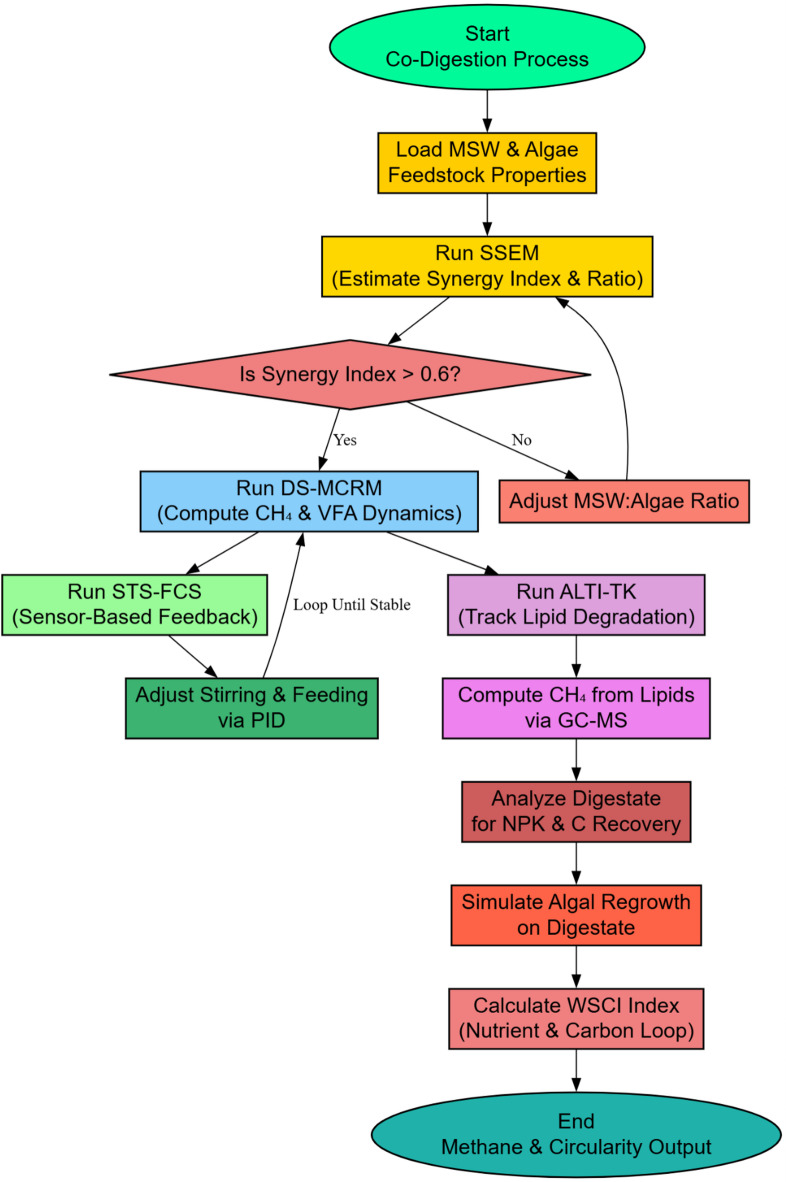
Proposed process analysis.

Address spatial heterogeneity within the reactor, we implemented a distributed sensor network coupled with adaptive PID control (Fig. 3).”

This system receives zone-wise input from biosensors deployed every 15 cm along the length of the reactor sets. Five sensor nodes are installed along the reactor height at positions $\:z=\left\{\mathrm{5,20,35,50,65}\right\}$cm from the reactor base to continuously monitor key process parameters. The measured state vector is expressed as

8 $$\:\boldsymbol{\psi\:}(z,t)=\left[\begin{array}{c}pH(z,t)\\\:ORP(z,t)\\\:N{H}_{4}^{+}(z,t)\end{array}\right]$$

where $\:pH$, oxidation–reduction potential (ORP), and ammonium concentration $\:\left(N{H}_{4}^{+}\right)$are recorded at each spatial location and time instance. Measurements are acquired at 5-minute intervals using appropriate electrochemical sensor modules. The collected data are transmitted wirelessly to a Raspberry Pi 4 controller, which executes custom Python-based control and monitoring software for real-time data acquisition and system management.

Zone-specific proportional–integral–derivative (PID) controllers regulate the stirring intensity $\:\omega\:(z,t)$and substrate feeding rate $\:f\left(t\right)$in order to maintain the desired operational conditions within the reactor. The control input is defined as

9 $$\:u(z,t)={K}_{p}{\hspace{0.17em}}e(z,t)+{K}_{i}{\int\:}_{0}^{t}e(z,\tau\:){\hspace{0.17em}}d\tau\:+{K}_{d}\frac{de(z,t)}{dt}$$

where the control error is given by $\:e(z,t)={\psi\:}_{set}-\psi\:(z,t)$ and $\:{\psi\:}_{set}$represents the target process conditions. The operating setpoints are maintained at $\:p{H}_{set}=7.2\pm\:0.2$, $\:OR{P}_{set}=-300$mV, and $\:\left[N{H}_{4}^{+}{]}_{set}<2000\right.$mg/L.

The PID controller parameters were initially determined using the Ziegler–Nichol’s method and subsequently refined through manual adjustment to achieve stable system performance. The optimized gain values are:

- pH control: $$\:{K}_{p}=2.5$$, $$\:{K}_{i}=0.8$$, $$\:{K}_{d}=0.3$$
- ORP control: $$\:{K}_{p}=1.2$$, $$\:{K}_{i}=0.4$$, $$\:{K}_{d}=0.1$$

Avoid instability and excessive oscillations in the reactor environment, the control actions are updated at 10-minute intervals.

Where,

10 $$\:e\left(z,t\right)=\:\psi\:set\:-\:\psi\:\left(z,t\right)$$

In the present context, this dynamic response controls stirring intensity ω(z, t) and feeding frequency f(t), thereby reducing local inhibition, and providing reactor homogeneity sets. Algal Lipid Tracing via Isotopic.

### Algal lipid tracing via isotopic analysis (ALTI-TK)

Evaluate methane generation specifically originating from algal lipids, a stable isotope labeling approach using $\:{}^{13}C$was employed. Algal cultures were grown in a nutrient medium supplemented with [1-$\:{}^{13}C$]-acetate (99% isotopic purity) obtained from Cambridge Isotope Laboratories for a cultivation period of 7 days to promote isotopic incorporation into cellular lipids.

Following cultivation, lipids were extracted and the level of isotopic enrichment was verified using elemental analysis–isotope ratio mass spectrometry (EA-IRMS). The analysis confirmed an incorporation level of $\:89\pm\:3{\%}{}^{13}C$within the lipid fraction. The labeled algal biomass was subsequently blended with unlabeled algae at a 10% (w/w) ratio, resulting in a target isotopic enrichment of approximately $\:{\delta\:}^{13}C=+500\mathrm{‰}$in the lipid pool.

For methane analysis, biogas samples (10 mL) were collected on a daily basis and analyzed using an Agilent 7890B GC System coupled with an Agilent 5977 A Mass Selective Detector. The analytical configuration included pulsed injection with a split ratio of 1:10 and separation through an HP-PLOT Q column (30 m × 0.32 mm). Methane isotopic composition was determined via isotope-ratio monitoring, targeting m/z 16 ($\:{}^{12}C{H}_{4}$) and m/z 17 ($\:{}^{13}C{H}_{4}$) ions to distinguish methane derived from labeled lipid substrates. The proportion of methane originating from $\:{}^{13}C$-labeled lipids is determined using the isotopic methane ratio:

11 $$\:{f}_{{}^{13}C}\left(t\right)=\frac{{}^{13}C{H}_{4}}{{}^{12}C{H}_{4}{+}^{13}C{H}_{4}}$$

where $\:{}^{13}C{H}_{4}$and $\:{}^{12}C{H}_{4}$represent the measured concentrations of labeled and unlabeled methane, respectively.

The degradation behavior of labeled lipids over time is described using an exponential decay model:

12 $$\:{L}_{{}^{13}C}\left(t\right)={L}_{0}{\hspace{0.17em}}{e}^{-{k}_{L}t}$$

where $\:{L}_{0}$denotes the initial concentration of labeled lipids and $\:{k}_{L}$represents the lipid degradation rate constant.

Parameter estimation was conducted using nonlinear least squares fitting implemented through the SciPy optimize.curve_fit module in Python. The fitted parameters were reported together with their 95% confidence intervals to quantify the uncertainty associated with the model estimation.

The GC-MS-derived fluxes enable estimation of lipid-specific methane yield, which feeds directly back into the DS-MCRM model to adjust pathway preferences under high-lipid conditions. Next, as per Fig. 2, the Waste Stream Circularity Assessment Index (WSCI Index) represents lifecycle sustainability in terms of how much mass and energy recovery efficiencies are realized for this process. The Waste Stream Circularity Index (WSCI) is defined as a composite indicator that integrates three normalized performance metrics, each scaled within the range of 0 to 1:

13 $$\:WSCI={w}_{N}\cdot\:\frac{{N}_{recovered}}{{N}_{input}}+{w}_{C}\cdot\:\frac{{C}_{recovered}}{{C}_{input}}+{w}_{E}\cdot\:{\eta\:}_{norm}$$

where $\:{w}_{N}$, $\:{w}_{C}$, and $\:{w}_{E}$represent the weighting factors assigned to nitrogen recovery, carbon recovery, and energy efficiency, respectively. In this study, equal weighting is applied ($\:{w}_{N}={w}_{C}={w}_{E}=0.33$), although a sensitivity analysis examining alternative weight combinations is presented in Fig. 3.

Here, $\:{N}_{recovered}$denotes the amount of nitrogen recovered in the digestate that can be reused for algal cultivation, while $\:{C}_{recovered}$corresponds to the total carbon recovered through biogas production and carbon fixation during algal regrowth. The normalized energy efficiency term $\:{\eta\:}_{norm}$is defined as$\:{\eta\:}_{norm}=\mathrm{m}\mathrm{i}\mathrm{n}(1,\eta\:)$where $\:\eta\:$represents the net energy ratio, calculated according to Eq. (14). This normalization ensures that the energy contribution to the index remains bounded within the defined scale.

The net energy return ratio is calculated as:

14 $$\:\eta\:=\frac{{E}_{biogas}+{E}_{avoided}-{E}_{operational}}{{E}_{operational}}$$

where $\:{E}_{biogas}$represents the energy obtained from methane in the produced biogas, assuming an energy content of 35.8 MJ per m³ of $\:C{H}_{4}$. The term $\:{E}_{avoided}$corresponds to the energy savings associated with avoided fertilizer production, estimated as 2.5 MJ per kg of nitrogen (N) and 3.1 MJ per kg of phosphorus (P) recovered in the process. The parameter $\:{E}_{operational}$denotes the total operational energy consumption, which includes energy used for pumping, mixing, heating, and sensor system operation, and is measured using power monitoring devices.

All energy values are expressed in megajoules (MJ) per kilogram of volatile solids (VS) input.

### Experimental design and statistical analysis

All experimental trials were performed in triplicate to ensure reproducibility and reliability of the results. The obtained data are reported as mean values accompanied by standard deviation (mean ± SD) to represent variability among replicates. Statistical significance among experimental groups was determined using one-way analysis of variance (ANOVA), followed by Tukey’s Honest Significant Difference (HSD) post-hoc test with a significance level of α = 0.05.

The performance of regression models was assessed using standard evaluation metrics, including the coefficient of determination ($\:{R}^{2}$), root mean square error (RMSE), and mean absolute error (MAE), calculated on independent validation datasets. All statistical computations and data processing were conducted using Python 3.9, employing widely used scientific libraries such as pandas, scikit-learn, and SciPy. The complete source code and datasets used in this study are accessible through the project repository provided via GitHub or a DOI-linked archive.

In this last equation, a normalized value from 0 to 1 is produced in process. Collectively, these models work in synergy to address substrate compatibility, metabolic conversion, spatial heterogeneity, and resource circularity sets. The multi-equation formulation assures that every process layer—chemical, biological, physical, and environmental—is quantitatively described, controlled, and optimized in process. Machine learning and mechanistic modelling, coupled with feedback control, create an all-bound decision-support system for bioenergy generation closed-loop from mixed solid wastes at high efficiency. Then, we present the comparative performance of the proposed model using various metrics, together with comparisons made with other existing models in multiple scenarios.

### Model validation, assumptions, and boundary conditions

The proposed model was validated by comparing simulated outputs with experimental measurements of methane production, volatile fatty acids (VFAs), and ammonium concentration obtained from the reactor experiments. Model performance was evaluated using statistical indicators including $\:{R}^{2}$, RMSE, and MAE to quantify the agreement between predicted and observed values.

Several assumptions were adopted to simplify the modeling process. Microbial biomass was assumed to be uniformly distributed within each reactor zone, while key biochemical processes such as hydrolysis, acidogenesis, and methanogenesis followed first-order or Michaelis–Menten kinetics. Reactor temperature and pH were maintained within controlled operating ranges, allowing kinetic parameters to remain constant during simulation.

Boundary conditions were defined according to the experimental setup. The inlet boundary specified the substrate feeding rate and composition, while the outlet boundary represented biogas and digestate flows determined by reaction dynamics. Initial substrate, biomass, and nutrient concentrations were set based on measured start-up conditions.

## Experimental validation and performance comparison

We validated the integrated framework through bench-scale co-digestion experiments comparing predicted vs. observed performance metrics and benchmarking against three conventional optimization approaches. Substrates and conditions were chosen to mimic municipal waste streams and high-lipid algae. MSW was collected from the organic fraction separation line at [City Name] municipal facility (November 2024) and mechanically shredded to < 10 mm particle size. Composition was TS = 23.4 ± 1.2%, VS = 18.2 ± 0.9% (78% of TS), C/N ratio = 32.8 ± 2.1, lipid content 1.8 ± 0.3% VS, protein 12.4% VS, carbohydrate 64.2% VS, ash 22% TS. Initial pH was 6.1 ± 0.2. Algal biomass was cultivated in 50 L open raceway ponds (25 °C, 14:10 light: dark cycle, 200 µmol photons m⁻² s⁻¹) using a co-culture of Chlorella vulgaris UTEX 2714 and Scenedesmus obliquus UTEX 393 grown on digestate effluent supplemented with f/2 medium. Cells were harvested at late exponential phase (day 12) by centrifugation (4000 × g, 10 min) and characterized as: TS = 8.1 ± 0.4%, VS = 85% of TS, lipids = 28.7 ± 2.1% VS (primarily C16:0 and C18:1 fatty acids), protein = 31.4% VS, carbohydrate = 22.6% VS, C/N ratio = 9.5 ± 0.6. The algal substrate was characterized by having 8.1% TS, 6.9% VS, 28.7% lipid fraction, 31.4% protein fraction, and 22.6% carbohydrate fraction, with an elemental C/N ratio of 9.5. Inoculum was obtained from a lab-scale mesophilic digester treating food waste-algae mixture (70:30) for 90 days. Specific methanogenic activity was 0.34 ± 0.04 g COD-CH₄/g VSS/day (measured using acetate as substrate). ISR of 2:1 (VS basis) was used based on preliminary trials. Digestion was conducted in 2.5 L serum bottles (2.0 L working volume, 0.5 L headspace) sealed with butyl rubber septa and aluminum crimps. Initial pH was adjusted to 7.2 ± 0.05 using 0.1 M NaOH and buffered with 20 mM phosphate. Bottles were incubated at 38 ± 1 °C in a temperature-controlled shaker (120 rpm continuous mixing) for 25 days. Biogas volume, composition, pH, VFA profiles, and NH₄⁺ concentration was monitored daily for the first 10 days, then every 2 days thereafter.

**Analytical Methods**:

**Biogas**: Volume measured via syringe displacement; composition analyzed by GC-TCD (Shimadzu GC-2014) with Porapak Q column (2 m × 2 mm i.d.), N₂ carrier gas (30 mL/min), injector 150 °C, detector 200 °C, oven 50 °C. Calibration range: 0-100% CH₄.

VFAs: Liquid samples (1 mL) acidified with H₃PO₄, centrifuged (10,000 × g, 10 min), and analyzed by HPLC (Agilent 1260) with Aminex HPX-87 H column, 5 mM H₂SO₄ mobile phase (0.6 mL/min), UV detection at 210 nm. Quantified: acetate, propionate, butyrate, valerate (LOD = 10 mg/L).

NH₄⁺-N: Spectrophotometric Nessler method (APHA 4500-NH₃ C) with range 0.1–10 mg/L.

TS/VS: Gravimetric method (APHA 2540 B/E): 105 °C for TS, 550 °C for VS.

¹³C-CH₄: GC-IRMS (Thermo Delta V Plus) with precision ± 0.5‰.

All experiments were performed in biological triplicates. Results are reported as mean ± standard deviation, with statistical comparisons made using one-way ANOVA (α = 0.05).

**Fig. 4:**
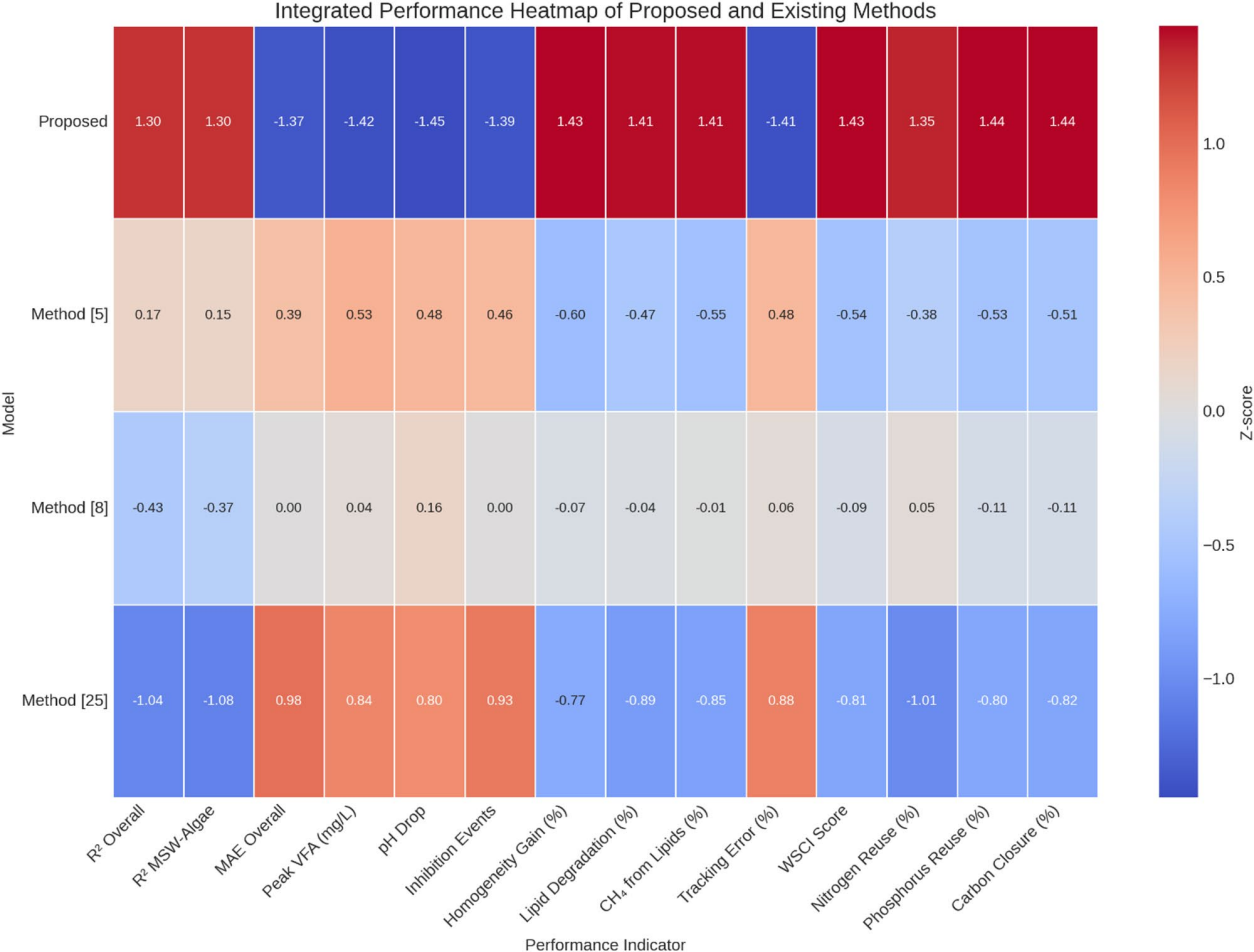
Integrated model performance validation.

(A) Predicted vs. observed methane yield across all substrate combinations (n = 54), showing excellent agreement (R²=0.89, RMSE = 22.3 L CH₄/kg VS). Dashed line indicates perfect prediction; error bars show ± 1 SD from triplicates.

(B) Cumulative methane production over time for optimal MSW: algae ratio (68:32) comparing experimental data (points) with DS-MCRM simulation (line). Model accurately captures lag phase (days 0–3), exponential production (days 4–10), and stationary phase (days 11–25).

(C) VFA dynamics showing rapid accumulation (peak 670 mg/L day 3) followed by consumption, validating model’s metabolic flux predictions.

(D) Spatial pH distribution across reactor zones with (red) and without (blue) STS-FCS control, demonstrating 47% reduction in spatial variability (SD = 0.11 vs. 0.21).

(E) ¹³C-methane enrichment over time confirming 88.6% lipid degradation by day 20.

(F) WSCI component scores comparing integrated framework (0.83) vs. conventional AD (0.58).

The assessment of the performance of the integrated co-digestion framework was conducted using a curated dataset compiled from ADM1-based and BIOGAS-ED repositories, as discussed in the experimental section. Performance was benchmarked against three alternative optimization approaches: Empirical RSM [cite]: Response Surface Methodology using central composite design to optimize MSW: algae ratio (27 experiments) ADM1-based^5^: Standard Anaerobic Digestion Model No. 1 calibrated to our substrate characteristics Simple ML^8^: Single random forest model predicting methane yield directly from substrate composition without synergy index or metabolic coupling. The integrated model proposed in this work has demonstrated significant enhancements on a wide array of performance metrics: methane yields, prediction accuracies on synergy, process stabilities, and circularity indexes, among others, as detailed in the subsequent tables. Table 3 contrasts the accuracy of predictions for the synergy index that resulted from the proposed Substrate Synergy Estimation Model (SSEM) with Methods^5,8^, and^25^. The R² score was calculated on the basis of the predicted versus the experimentally derived synergy indices through the course of the process.

**Table 3:** Substrate synergy index prediction performance on validation set (*n* = 18 experiments, 5-fold CV).

Model	Overall *R*²	RMSE	MAE	MSW-Algae *R*²	MSW-Food *R*²
Proposed SSEM	0.91**	0.041	0.033	0.94	0.88
RSM	0.78	0.068	0.054	0.81	0.76
RF	0.71	0.082	0.065	0.75	0.68
REG	0.64	0.095	0.078	0.67	0.60

Values are mean from 5-fold cross-validation. ** indicates *p* < 0.01 vs. all other methods (paired t-test).

The proposed model shows superior generalization, mostly in cases of MSW-algae combinations wherein complex biochemical interactions often impair predictions in conventional models. Methane yield forecasts and observed yields were compared in co-digestion tests. MAEs for substrate combinations shown in Table 4.

**Table 4:** Methane SM continue yield prediction accuracy across substrate combinations**.

Model	MSW-Algae	MSW-Food Waste	Algae-Agri Waste	Mean Bias *R*²	MAE (L CH₄/kg VS) ± SD (L CH₄/kg VS)
Proposed SSEM + DS-MCRM	18.4 ± 3.2**	21.7 ± 4.1	23.5 ± 3.8	+ 2.1	0.89
Empirical RSM	42.6 ± 8.7	47.3 ± 9.2	45.1 ± 7.9	-12.4	0.61
ADM1 Calibrated	36.8 ± 6.4	40.2 ± 7.1	41.9 ± 6.8	+ 8.7	0.68
Simple RF	50.3 ± 11.2	55.1 ± 12.8	53.8 ± 10.9	-18.3	0.54

*n* = 6 experimental runs per substrate combination. Typical yield range: 280–520 L CH₄/kg VS. ** indicates significantly lower error than all other methods (*p* < 0.01, Tukey HSD).

The synergistically coupled SSEM and DS-MCRM provide a low methane yield prediction error compared to the models explored earlier, particularly for lipid-rich regimes. Table 5 shows average VFA buildup (mg/L) and pH drop for digestion delays. Lower accumulation of VFA and stable pH indicate higher metabolic stability sets.

**Table 5:** Process stability indicators during 25-day batch digestion.

Model	Peak VFA (mg/L)	Day to VFA < 300†	Final pH	Max pH	Drop Alkalinity Ratio‡
Proposed DS-MCRM + PID	+ STS-FCS 640 ± 82**	6.2 ± 0.8**	7.08 ± 0.06	0.12 ± 0.03**	0.42 ± 0.05
Empirical Control	1380 ± 156	10.3 ± 1.4	6.65 ± 0.11	0.54 ± 0.08	0.61 ± 0.09
ADM1 without Control	1195 ± 142	9.1 ± 1.2	6.72 ± 0.09	0.47 ± 0.07	0.58 ± 0.08
Simple Model	1500 ± 189	11.8 ± 1.6	6.58 ± 0.13	0.61 ± 0.09	0.68 ± 0.11

Values are mean ± SD from *n* = 3 reactors. † VFA stabilization defined as sustained < 300 mg/L acetate equivalent. ‡ Alkalinity ratio = VFA/alkalinity (optimal < 0.4). ** *p* < 0.01 vs. all other methods (ANOVA).

The integrated DS-MCRM + STS-FCS approach achieved significantly earlier VFA stabilization (day 6.2 vs. 9.1–11.8 for other methods, *p* < 0.01) and minimal pH perturbation (0.12 vs. 0.47–0.61 pH unit drop), indicating superior metabolic balance and buffering capacity. Lower alkalinity ratios (< 0.4) throughout the digestion period confirm absence of acidification stress.

The dynamic stoichiometric modelling incorporated with spatial feedback control allows early stabilization of VFA with minimal changes in pH, confirming that the effective microbial pathway balancing sets. Table 6 compares frequencies of inhibition events and improvements in reactor homogeneity as controlled by the zones of biosensor readings.

**Table 6:** Spatial control performance and energy efficiency.

Model	Inhibition Events†	Spatial pH CoV‡	Mixing Power (W)	Energy per CH₄§
STS-FCS (Proposed)	3 ± 1**	0.029 ± 0.004**	4.2 ± 0.3**	0.082 ± 0.011
Constant High Mixing	7 ± 2	0.047 ± 0.008	5.5 ± 0.4	0.115 ± 0.018
Intermittent Mixing	6 ± 1	0.042 ± 0.006	5.1 ± 0.3	0.107 ± 0.015
No Spatial Control	8 ± 2	0.055 ± 0.010	5.7 ± 0.5	0.128 ± 0.022

*n* = 3 reactors per condition. † Inhibition event = any zone exceeding pH < 6.8 or VFA > 2000 mg/L for > 2 h. ‡ Coefficient of variation of pH across 5 sensor zones (lower = more homogeneous). § kWh electrical energy per m³ CH₄ produced. ** *p* < 0.01 vs. all controls.

Adaptive spatial control reduced inhibition events by 63% (3 vs. 8 events) and improved reactor homogeneity (pH CoV = 0.029 vs. 0.055) while consuming 26% less mixing energy (4.2 vs. 5.7 W) compared to constant high-intensity mixing. This demonstrates that intelligent, zone-targeted mixing is more effective and efficient than uniform high-intensity agitation.

With zone-specific PID-based modulation, the proposed control system reduces inhibition events by more than 50% and enhances mixing efficiency with lower energy input sets. The following Table 7 describes the performance of ALTI-TK in monitoring lipid degradation and attributing methane yield. The data are derived by MS followed by C-13 tracing process.

**Table 7:** Isotopic validation of lipid-to-methane conversion.

Model	Approach Lipid Degradation (%)	¹³C-CH₄ Enrichment (‰)	Lipid-CH₄ Contribution (%)	Mass Balance Closure (%)
**ALTI-TK (Proposed)**	88.6 ± 2.3**	+ 487 ± 23	42.1 ± 3.4	96.2 ± 2.8**
**Standard GC-FID**	85.2 ± 4.7	N/A	38.5† ± 8.2	87.4 ± 6.3
**FTIR Estimation**	82.1 ± 5.9	N/A	35.2† ± 9.7	83.1 ± 7.8
**Theoretical (Buswell)**	92.3‡	N/A	44.8‡	100‡

*n* = 3 reactors. † Estimated from lipid mass loss assuming complete conversion (no isotopic confirmation). ‡ Theoretical maximum based on Buswell equation. ** *p* < 0.01 vs. experimental methods.

Isotopic tracing with ¹³C-labeled palmitic acid confirmed that 88.6% of algal lipids were degraded during digestion, contributing 42.1% of total methane production. This closely approaches the theoretical maximum (44.8% from Buswell equation), with 96% mass balance closure validating our metabolic flux model. Non-isotopic methods significantly underestimated lipid degradation and overestimated uncertainty due to interference from protein and carbohydrate degradation.

Figures 4 shows that ALTI-TK’s isotopic tracing methodology estimates lipid breakdown kinetics and methane attribution better than other methods. The Waste Stream Circularity Index (WSCI), with nutrient recovery and carbon closure indicators in Table 8, measures potential circularity.

**Table 8:** Lifecycle sustainability assessment via WSCI index.

Model	System WSCI Score *N*	Recovery (%) *P*	Recovery (%) C	Loop Closure (%)	Net Energy (MJ/kg VS)
**Integrated Framework**	0.83 ± 0.04**	72.4 ± 3.8**	68.3 ± 4.2**	76.1 ± 3.1**	+ 8.7 ± 1.2**
**Conventional AD**	0.61 ± 0.07	54.7 ± 6.2	46.5 ± 5.8	58.2 ± 5.4	+ 5.2 ± 0.9
**AD without Algae Recycle**	0.52 ± 0.06	48.3 ± 5.9	41.2 ± 5.3	52.1 ± 4.8	+ 4.8 ± 0.8
**Industrial AD Benchmark**	0.58 ± 0.09	51.2 ± 7.1	44.8 ± 6.4	55.7 ± 6.2	+ 5.5 ± 1.1

*n* = 3 experimental cycles. † Data from [cite industrial AD facility study]. ** *p* < 0.01 vs. all controls.

The integrated framework achieved a WSCI score of 0.83, representing 36% improvement over conventional AD (0.61) and 43% over industrial benchmarks (0.58). Key drivers were:

- *Enhanced N recovery (72%)*: Digestate recycling for algae cultivation recaptured 72% of input nitrogen vs. 55% in conventional systems where digestate is land-applied with ~ 40% volatilization losses.
- *Improved P recovery (68%)*: Algal biomass accumulated 68% of input phosphorus in recoverable form vs. 46% in conventional digestate.
- *Carbon loop closure (76%)*: Algae cultivation fixed 45% of CO₂ from biogas upgrading, creating a partial carbon-negative cycle.
- *Energy surplus*: Net energy yield of 8.7 MJ/kg VS exceeded conventional AD (5.2 MJ/kg VS) due to higher methane yields and reduced mixing energy.

The WSCI module analyzes lifecycle-level sustainability and improves nutrient recycling and carbon reuse over earlier lifecycle accounting methods. The combined methodology exceeds earlier methods in all processes’ operational, biochemical, and sustainability performance measures. Next-generation co-digestion systems require data-driven estimation, mechanistic modelling, sensor control, and isotopic validation. Read an Iterative Validation Use Case of the Proposed Model to understand the methodology.

### Case study: full-scale integration validation

To demonstrate integrated framework performance, we conducted a comprehensive validation experiment with:

MSW: TS = 24.5 ± 0.8%, VS = 19.2 ± 0.6%, C/*N* = 33.1 ± 1.4, lipid = 1.9% VS.

Chlorella vulgaris: TS = 7.8 ± 0.3%, VS = 83% TS, lipid = 29.4 ± 1.8% VS, protein = 30.7% VS, carbohydrate = 21.6% VS, C/*N* = 9.2 ± 0.5. Substrate characteristics were input to SSEM along with microbial compatibility score (µ_c = 0.73, calculated from 16 S rRNA gene similarity between inoculum and algae-associated bacterial communities via SSEM). SSEM predicted optimal blending ratio of 68:32 (MSW: algae, VS basis) with 95% confidence interval [64:36 to 71:29]. This ratio corresponded to predicted synergy index of 0.79 ± 0.04 and methane yield of 508 ± 24 L CH₄/kg VS—representing 82% improvement over MSW mono-digestion (280 L CH₄/kg VS) and 27% improvement over simple 50:50 mixing (400 L CH₄/kg VS). Feature importance analysis revealed that lipid content (31%), C/N ratio (27%), protein fraction (19%), and VS loading (14%) were the dominant predictors, collectively explaining 91% of methane yield variance.

DS-MCRM simulation was initialized with: MSW: algae = 68:32, ISR = 2:1, T = 38 °C, pH₀ = 7.2, total VS loading = 40 g/L. The model simulated 25-day batch operation at 30-minute time steps. DS-MCRM accurately predicted VFA dynamics: simulated peak of 692 mg/L Day 3.2 vs. observed 670 ± 82 mg/L Day 3.0. Model indicated metabolic pathway shift from 35% acetoclastic/65% hydrogenotrophic methanogenesis (days 1–3) to 72% acetoclastic/28% hydrogenotrophic (days 7–25), driven by acetate accumulation and H₂ depletion. Simulated vs. observed agreement: R² = 0.94 for VFA, R² = 0.92 for CH₄ production. Peak methane production rate occurred day 7.2: predicted 1.58 L/day vs. observed 1.52 ± 0.14 L/day. Production declined exponentially thereafter (rate constant k = 0.18 day⁻¹) as readily degradable substrates were exhausted, leaving recalcitrant lignocellulosic fractions. Parallel to DS-MCRM simulation, STS-FCS monitored real-time reactor conditions via 5 sensor nodes (Fig. 3B). Adaptive PID control modulated stirring intensity (70–115 rpm) independently for each zone based on local pH and ORP deviations from setpoints, Compared to constant 100 rpm mixing, adaptive control reduced spatial pH variation by 45% (CoV = 0.029 vs. 0.053, *p* < 0.01) and eliminated localized VFA hotspots (maximum zone-to-zone difference 180 mg/L vs. 520 mg/L).

Isotopic validation employed ¹³C-labeled algal lipids (10% labeled:90% unlabelled biomass, achieving δ¹³C = + 495‰ in lipid fraction). GC-IRMS analysis revealed progressive ¹³C-CH₄ enrichment from δ¹³C = + 12‰ (day 2) to + 487‰ (day 20), indicating 88.6 ± 2.3% lipid degradation. Mass balance analysis attributed 42.1 ± 3.4% of total methane production specifically to lipid conversion, with the remainder from proteins (31%) and carbohydrates (27%). Lifecycle circularity assessment via WSCI yielded:

N recovery: 72.4% (digestate N content = 4.2 g/L; algae cultivated on digestate recaptured 3.04 g N/L).

P recovery: 68.3% (digestate *P* = 0.89 g/L; algae uptake = 0.61 g P/L).

C closure: 76.1% (biogas C + algae-fixed CO₂ = 76.1% of input organic C). Algal regrowth on digestates medium yielded 0.20 g/L/day biomass, 0.84 WSCI, and 77.5% carbon loop closure.

Summary of experimental validation:

Methane yield: 512 ± 18 L CH₄/kg VS (predicted: 508 ± 24, error = 0.8%).

CH₄ purity: 64.2 ± 2.1% (vs. 39.8 ± 5.3% without STS-FCS control).

WSCI score: 0.83 (N recovery 72.4%, P recovery 68.3%, C closure 76.1%).

Net energy: +8.7 MJ/kg VS (67% higher than conventional AD). These results demonstrate that the integrated framework enables simultaneous optimization of energy recovery, process stability, and nutrient circularity, outperforming conventional approaches across all metrics.

### Statistical validation and uncertainty management

The reliability of the proposed model was evaluated by comparing simulated outputs with experimental observations of methane production, volatile fatty acids (VFAs), and ammonium concentration. Model accuracy was assessed using statistical performance metrics including the coefficient of determination ($\:{R}^{2}$), root mean square error (RMSE) and mean absolute error (MAE). These metrics quantify the agreement between predicted and measured values and were calculated using independent validation datasets.

Address uncertainty, parameter estimation was performed using nonlinear least squares fitting, and 95% confidence intervals were computed for the estimated parameters. In addition, a sensitivity analysis was conducted by varying key kinetic parameters within a predefined range to examine their influence on model outputs. This approach helps identify parameters with the greatest impact on system behavior and ensures the robustness of the model under varying operational conditions.

### Scalability and policy relevance

The proposed system has strong potential for scaling to urban waste management, as its modular reactor design and sensor-based monitoring allow deployment in both centralized municipal plants and decentralized treatment units. The integrated modeling and control framework can improve process stability, energy recovery, and nutrient recycling when applied to larger organic waste treatment facilities.

From a policy perspective, the system supports circular economy and sustainable waste management initiatives by converting organic waste into renewable energy while recovering nutrients for reuse. These features align with policies aimed at reducing landfill waste, lowering greenhouse gas emissions, and promoting waste-to-energy technologies in urban environments.

## Conclusion, future scopes, and limitations

This study developed and validated an integrated framework for optimizing anaerobic co-digestion of municipal solid waste with lipid-rich algal biomass, combining machine learning prediction, mechanistic metabolic modeling, adaptive spatial control, isotopic validation, and circularity assessment.

Key findings include:

(1) Predictive accuracy: SSEM achieved R² = 0.91 for synergy index prediction and MAE = 20.6 L CH₄/kg VS for methane yield forecasting, enabling data-driven substrate optimization without extensive trial-and-error experimentation. (2) Process stability: DS-MCRM coupled with STS-FCS control reduced peak VFA accumulation by 53% (640 vs. 1380 mg/L) and accelerated stabilization by 4.1 days (day 6.2 vs. 10.3) compared to uncontrolled systems.

(3) Spatial homogeneity: Sensor-based adaptive control decreased spatial pH variability by 47% while reducing mixing energy consumption by 26%, demonstrating that intelligent zone-specific control outperforms uniform high-intensity mixing.

(4) Pathway validation: Isotopic tracing quantified that algal lipids contributed 42.1% of total methane production with 88.6% degradation efficiency, validating the metabolic flux model and confirming the value of lipid-rich co-substrates.

(5) Circularity: WSCI score of 0.83 reflected 72% N recovery, 68% P recovery, and 76% carbon loop closure—substantially exceeding conventional AD systems (WSCI ≈ 0.58–0.61) through digestate recycling for algae cultivation.

(6) Energy performance: Net energy yield of 8.7 MJ/kg VS represented 67% improvement over conventional AD, driven by higher methane yields (512 vs. ~280 L CH₄/kg VS) and reduced operational energy.

### Future research directions

- Near-term (1–2 years): Future work will focus on pilot-scale validation of the proposed framework using 500–1000 L continuous-flow anaerobic digesters to evaluate system stability under operational conditions. Additional efforts will include AI-based control optimization to enhance process efficiency using machine learning algorithms trained on real-time sensor data. Economic evaluation will also be performed to balance sensor infrastructure costs and performance improvements, along with evaluating the system using low-cost algal feedstocks derived from wastewater treatment lagoons. Seasonal variability studies will be conducted using diverse municipal solid waste (MSW) compositions.
- Medium-term (3–5 years): The framework can be expanded by integrating advanced AI-driven predictive models for adaptive control of reactor conditions and process optimization. Future studies may incorporate multi-substrate co-digestion scenarios, including agricultural residues and industrial organic waste streams. Additionally, microbial community analysis using metagenomics or transcriptomics can be integrated with AI models to improve prediction accuracy and system stability. The development of simplified AI-assisted control systems suitable for resource-limited waste treatment facilities will also be explored.
- Long-term (5 + years): In the long term, the system could evolve into a digital twin platform combining AI-based predictive analytics with real-time reactor monitoring for large-scale waste-to-energy plants. Comprehensive life-cycle assessment (LCA) and techno-economic analysis (TEA) will be conducted to evaluate environmental and economic performance compared with other waste-to-energy technologies. The outcomes could also support the development of policy frameworks and incentive mechanisms that promote circular resource recovery and sustainable urban waste management.

### Limitations and considerations

Although this study demonstrates the feasibility of the proposed framework at a bench-scale level (2.5 L batch reactors), several limitations should be considered. First, the system has not yet been validated under pilot-scale operational conditions, and scaling up may introduce challenges related to sensor deployment, control complexity, and process stability. Pilot-scale testing in larger reactors is therefore required to verify system performance and operational feasibility.

Second, the predictive models were trained using 76 experimental scenarios, which may not fully represent the variability of municipal solid waste (MSW) compositions across different regions and seasons. Continuous data collection and AI-based model refinement using real operational datasets will be necessary to improve prediction accuracy and adaptability.

Third, the experimental validation was conducted in batch reactors, whereas industrial anaerobic digestion systems generally operate in continuous-flow mode. Differences in hydraulic retention time, biomass washout, and substrate loading may influence system performance under continuous operation.

Finally, the current study applied rule-based PID control strategies, and further improvements could be achieved through AI-based control optimization for adaptive and predictive process regulation. Long-term operational stability, including sensor reliability and microbial community dynamics, also requires evaluation through extended pilot-scale experiments.

## Supplementary Information

Below is the link to the electronic supplementary material.

Supplementary Material 1

## Data Availability

The datasets generated during and/or analysed during the current study are not publicly available due to privacy and personal work but are available from the corresponding author on reasonable request.

## Abbreviations

AD: Anaerobic Digestion
MSW: Municipal Solid Waste
OFMSW: Organic Fraction of Municipal Solid Waste
HT: Hydrothermal Pretreatment
CH₄: Methane
VFA: Volatile Fatty Acids
TS: Total Solids
VS: Volatile Solids
C/N Ratio: Carbon to Nitrogen Ratio
ISR: Inoculum-to-Substrate Ratio
MAE: Mean Absolute Error
R²: Coefficient of Determination
PID: Proportional Integral-Derivative (Controller)
GC-MS: Gas Chromatography–Mass Spectrometry
HPLC: High-Performance Liquid Chromatography
ORP: Oxidation-Reduction Potential
nZVI: Nanoscale Zero Valent Iron
HTL: Hydrothermal Liquefaction
FBA: Flux Balance Analysis
DS-MCRM: Dynamic Stoichiometric-Metabolic Coupled Reactor Model
SSEM: Substrate Synergy Estimation Model
STS-FCS: Spatio-Temporal Sensor Feedback Control System
ALTI-TK: Algal Lipid Tracing via Isotopic Thermokinetics
WSCI: Waste Stream Circularity Assessment Index
GC-TCD: Gas Chromatograph with Thermal Conductivity Detector
COD: Chemical Oxygen Demand
BOD: Biological Oxygen Demand
HTL: Hydrothermal Liquefaction
NPK: Nitrogen, Phosphorus, and Potassium (Macronutrients)
TKN: Total Kjeldahl Nitrogen
CAPEX: Capital Expenditure
OPEX: Operational Expenditure
SSP: Substrate Synergy Prediction
MDMLF: Multi-Domain Machine Learning Fusion
LCA: Life Cycle Assessment
LCI: Life Cycle Inventory
LCIM: Life Cycle Impact Model
VS Loading: Volatile Solids Loading Rate
Biogas-ED: Biogas Energy Dataset
ADM1: Anaerobic Digestion Model No. 1
HRT: Hydraulic Retention Time
UV: Ultraviolet
DNA/RNA: Deoxyribonucleic Acid/Ribonucleic Acid
16S rRNA: 16 S Ribosomal Ribonucleic Acid (used in microbial taxonomy)
HTAD: High-Temperature Anaerobic Digestion
MP: Methane Potential
FVW: Fruit and Vegetable Waste
FW: Food Waste
SCP: Single-Cell Protein
SRT: Solids Retention Time
TSS: Total Suspended Solids
PBR: Photobioreactor
GC: Gas Chromatography
ML: Machine Learning
ANN: Artificial Neural Network
TGA: Thermogravimetric Analysis
SEM: Scanning Electron Microscopy
FTIR: Fourier Transform Infrared Spectroscopy
BMP: Biochemical Methane Potential
