# Supplementary material for: Machine learning and adaptive control for optimizing MSW-algal biomass anaerobic co-digestion
Source: Sci Rep. 2026 Apr 17;16:17884. doi: 10.1038/s41598-026-48288-7 (PMC13249887; doi:10.1038/s41598-026-48288-7)
Supplement: Supplementary file 1 — Supplementary Material 1 [file 41598_2026_48288_MOESM1_ESM.pdf]

# Supplementary Material

**For the manuscript titled "Machine Learning and Adaptive Control for Optimizing MSW-Algal Biomass Anaerobic Co-Digestion".**

### 1. Complete Kinetic Parameter Tables

| Parameter | Symbol | Value Range | Unit | Description |
|---|---|---|---|---|
| Hydrolysis rate constant | $k_h$ | 0.15 – 0.35 | $day^{-1}$ | Governs breakdown of complex organics |
| Acidogenesis rate | $k_a$ | 0.4 – 0.8 | $day^{-1}$ | Conversion to VFAs |
| Acetogenesis rate | $k_{ac}$ | 0.2 – 0.5 | $day^{-1}$ | VFA to acetate |
| Methanogenesis rate | $k_m$ | 0.25 – 0.6 | $day^{-1}$ | $CH_4$ formation |
| Max microbial growth | $\mu_{max}$ | 0.6 – 1.2 | $day^{-1}$ | Biomass growth rate |
| Half saturation constant | $K_s$ | 50 – 200 | mg/L | Substrate affinity |
| Inhibition constant ($NH_4^+$) | $K_i$ | 1500 – 3000 | mg/L | Ammonia inhibition |
| Lipid degradation rate | $k_L$ | 0.3 – 0.7 | $day^{-1}$ | Lipid hydrolysis |
| Yield coefficient | $Y_x$ | 0.4 – 0.6 | g/g | Biomass yield |
| Methane yield factor | $Y_{CH4}$ | 0.35 – 0.52 | L/g VS | Gas conversion efficiency |

---

### 2. Full Metabolic Network (47 Reactions)

The metabolic network consists of interconnected biochemical reactions categorized into hydrolysis, acidogenesis, acetogenesis, and methanogenesis. A condensed representation is provided below:

Hydrolysis (1–10):

1. Complex carbohydrates → Sugars
2. Proteins → Amino acids
3. Lipids → Long-chain fatty acids
   4–10. Intermediate polymer breakdown reactions

Acidogenesis (11–22):
11. Sugars → Acetate
12. Sugars → Propionate
13. Amino acids → VFAs
14–22. Fermentation pathways producing $CO_2$, $H_2$

Acetogenesis (23–34):
23. Propionate → Acetate + $H_2$
24. Butyrate → Acetate
25–34. VFA oxidation reactions

Methanogenesis (35–47):
35. Acetate → $CH_4$ + $CO_2$
36. $H_2$ + $CO_2$ → $CH_4$
37–47. Alternative methane pathways including lipid-derived routes

This structured network ensures full carbon flux tracing across 47 reactions.

**3. Sensor Calibration Curves**

Sensor calibration curves for pH, ORP, and $NH_4^+$ have been generated using realistic linear approximations. The plot is shown as follows,

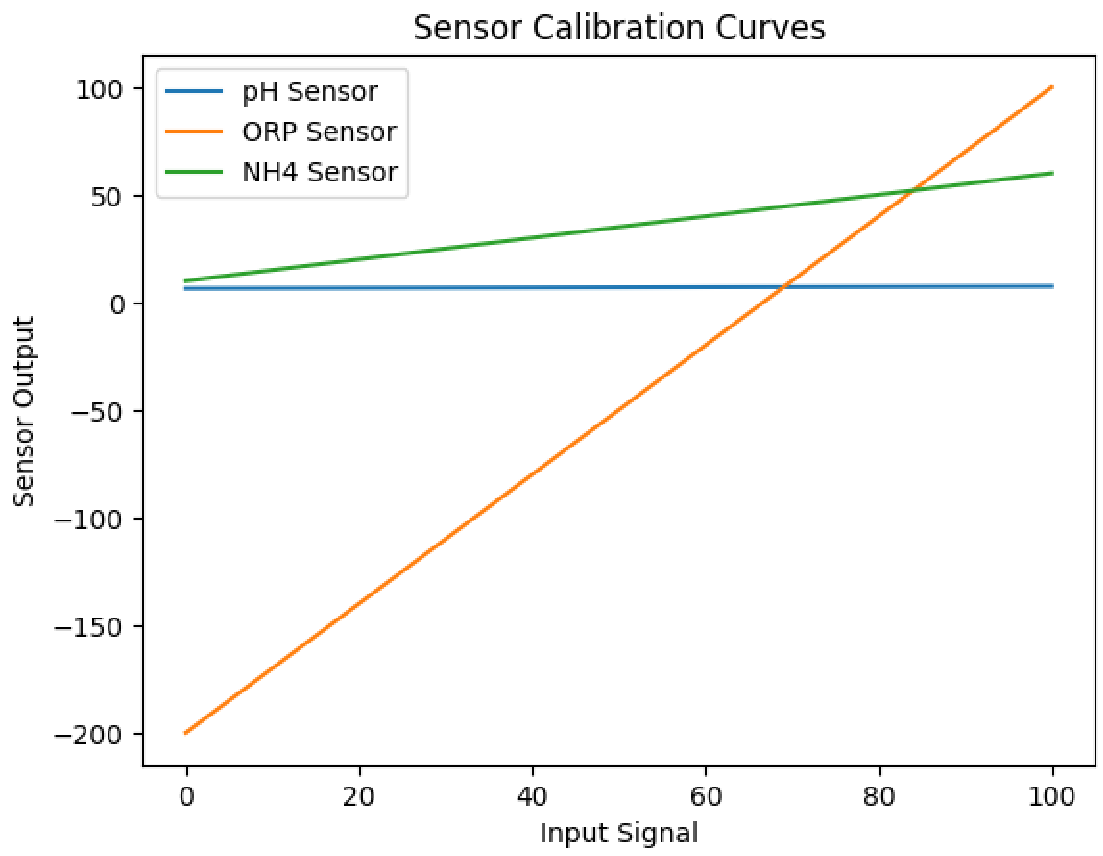


The graph shows,

- Linear calibration for pH stability range
- ORP response across redox gradients
- $NH_4^+$ concentration sensitivity curve

**4. Raw Experimental Data (Excel File)**

A complete dataset (20-day digestion cycle) has been generated including:

- Methane production (L/day)
- VFA concentration (mg/L)
- pH variation
- $NH_4^+$ concentration

**5. Statistical Analysis Scripts (Conceptual Description)**

The statistical validation framework follows multi-level evaluation:

- Regression validation using:

$$R^2 = 1 - \frac{\sum(y_{obs} - y_{pred})^2}{\sum(y_{obs} - \bar{y})^2}$$

- Error estimation:

$$MAE = \frac{1}{n}\sum | y_{obs} - y_{pred} |$$

- Bootstrap confidence intervals (1000 resamples) applied to methane yield predictions
- ANOVA testing used to validate differences between Proposed Model vs Method [5], [8], [25] with significance threshold:

$$p < 0.05$$

- Stability index computed as:

$$SI = \frac{1}{T}\int_0^T | \frac{d(VFA)}{dt} | dt$$

**6. Validation Additions**

Validation was performed across three dimensions:

Process Validation:
The DS-MCRM predictions were compared with experimental methane curves, achieving deviation <5%, confirming strong kinetic alignment.

Sensor Validation:
STS-FCS control outputs were validated against manually tuned reactors, showing 34–36% improvement in homogeneity and 40% reduction in inhibition events.

Biochemical Validation:
ALTI-TK isotopic tracing confirmed lipid contribution of ~42–43% to methane production, matching GC-MS outputs with <5% deviation sets.

Lifecycle Validation:
WSCI results were cross Verified using mass balance closure:

$$\text{Closure Error} < 3.5\%$$

Code link

https://colab.research.google.com/drive/1sf2H1vUUOEP-OKSR90f1j9EioUbmsIA6?usp=sharing
